# Strategic value driven by artificial intelligence in global businesses: a bibliometric and qualitative analysis of the most influential literature

**DOI:** 10.3389/frai.2026.1800412

**Published:** 2026-04-13

**Authors:** Melva Jenny Zambonino-Torres, Jaime Marcelo Coello-Viejó, Silvia Carolina Zambonino-Torres

**Affiliations:** State University of Milagro, Milagro, Ecuador

**Keywords:** algorithmic governance, competitive advantage, digital transformation, dynamic capabilities, explainability (XAI)

## Abstract

Gender equity remains a multidimensional and persistent challenge despite significant advances in public policy, health systems, labor structures, and sociocultural transformation. Existing research often approaches gender equity from fragmented perspectives, limiting a comprehensive understanding of its structural determinants and policy implications. This study addresses this gap by adopting an interdisciplinary approach to analyze how gender equity is conceptualized and studied within public policy-related domains. This study employs a mixed methodological design combining bibliometric analysis and systematic literature review. Scientific production was retrieved from Scopus and Web of Science using structured search equations applied to titles, abstracts, and keywords. Inclusion and exclusion criteria ensured thematic relevance and methodological rigor. The dataset was cleaned and processed using R, including duplicate removal and keyword-based filtering. Bibliometric techniques were applied to identify productivity patterns, citation impact, and thematic clusters, while a qualitative synthesis of selected influential studies provided deeper interpretive insights. The findings reveal a significant growth in scientific production on gender equity, with a strong concentration in health, labor, and social policy domains. Thematic analysis identified key clusters related to structural inequality, care systems, labor participation, and sociocultural norms. However, the results also highlight persistent gaps, particularly in the integration of interdisciplinary perspectives and the limited representation of Global South contexts. Additionally, the literature shows an imbalance between descriptive approaches and the development of actionable policy frameworks. The study demonstrates that gender equity research is evolving toward greater conceptual and methodological complexity but remains fragmented across disciplines. The dominance of certain regions and thematic areas suggests structural inequalities in knowledge production. These findings underscore the need for integrative frameworks that connect public policy, health, labor, and sociocultural dimensions to advance more effective and inclusive strategies. The study contributes to the field by offering a comprehensive mapping of research trends and identifying critical gaps that can inform future research and policy design.

## 1 Introduction

The accelerated development of artificial intelligence (AI) technologies—including machine learning, explainable artificial intelligence (XAI), and, more recently, generative models—has profoundly transformed the ways in which organizations create value, formulate strategies, and manage knowledge-intensive processes. Across diverse industries, AI systems are increasingly embedded in decision-support tools, operational optimization systems, predictive analytics platforms, and novel digital business models. Consequently, AI is progressively regarded not merely as a technological innovation but as a strategic resource capable of fundamentally reshaping competitive dynamics and organizational capabilities in contemporary business environments.

In knowledge-intensive services and digital markets, AI enables the processing of large-scale datasets, enhances predictive decision-making, and supports innovation processes that allow firms to respond more swiftly to complex and turbulent environments. These capabilities are especially pertinent in sectors characterized by high informational intensity, where algorithmic systems underpin managerial decisions, automate routine processes, and facilitate more advanced forms of customer engagement. Nevertheless, the deepening integration of AI also gives rise to substantial challenges concerning governance, transparency, algorithmic bias, digital competencies, and user/customer acceptance of automated decision systems (Bandara et al., 2025; Erdmann and Toro-Dupouy, 2025; Vorobeva et al., 2025). These tensions underscore the necessity of examining AI through organizational, institutional, and strategic lenses, in addition to its purely technological dimensions.

The influence of AI extends well beyond inherently digital sectors. Its applications encompass intelligent manufacturing, logistics, supply chains, agribusiness, energy systems, financial services, and numerous other domains. Within these contexts, AI intersects with broader structural transformations, including the digital transition and the socio-environmental transition, frequently framed within the paradigms of Industry 4.0 and the emerging Industry 5.0 paradigm (De Felice et al., 2025; Festa et al., 2025; Torrent-Sellens et al., 2025). In manufacturing and production systems, AI facilitates process optimization, predictive maintenance, and the development of digital twins that support sophisticated simulations and more efficient engineering workflows. These advancements enable organizations to enhance operational efficiency while simultaneously incorporating human-centric and sustainability-oriented principles into technological transformation.

In service industries and marketing contexts, recent empirical evidence indicates that AI—particularly generative AI—can augment creativity, productivity, and decision quality when its adoption is supported by appropriate organizational structures, leadership commitment, and institutional preparedness. Such dynamics are frequently analyzed through frameworks such as the Technology–Organization–Environment (TOE) model and related perspectives on digital innovation (Wang and Zhang, 2025b). Concurrently, research shows that the manner in which organizations communicate their AI capabilities significantly shapes customer perceptions and willingness to engage with automated services. For example, an overemphasis on algorithmic superiority may erode trust when customers anticipate human interaction or empathetic service delivery (Vorobeva et al., 2025). These insights reveal the intricate interplay between technological innovation and social acceptance in AI-enabled service ecosystems.

Parallel trends are observable in financial digitalization and cybersecurity. Emerging approaches integrate artificial intelligence with behavioral science techniques—such as gamification and personalization—to bolster risk mitigation strategies and enhance user engagement in digital financial environments. Yet, the rapid evolution of digital infrastructures simultaneously introduces novel vulnerabilities and threat vectors, necessitating adaptive governance mechanisms and more robust regulatory frameworks (Shahzadi et al., 2025). Organizations thus increasingly confront the challenge of reconciling innovation imperatives with risk management and ethical responsibility.

Beyond intra-organizational processes, AI also shapes broader socio-economic and environmental dynamics. In environmental policy and sustainability management, for instance, AI-driven analytical tools enable researchers and policymakers to investigate complex interrelationships among digitalization, industrial activity, and environmental outcomes. Recent studies employing interpretable machine learning methods, such as SHAP analysis, have uncovered nonlinear associations between digital transformation and carbon emissions, yielding valuable implications for the formulation of low-carbon policies and climate strategies (Wang S. et al., 2025). At the macroeconomic level, evidence suggests that digital maturity and fintech innovation may contribute to more sustainable environmental trajectories in advanced economies (Teng et al., 2025). In emerging economies, AI-enabled green innovation is increasingly associated with sustainable growth and accelerated energy transitions aligned with the Sustainable Development Goals (Behera et al., 2025).

Within organizational settings, AI integration also reconfigures internal capabilities and workforce dynamics. In B2B contexts, research on key account management indicates that personality traits, leadership styles, and organizational culture moderate AI technology adoption and mediate its effects on firm performance and competitive advantage (Mehta et al., 2025). Likewise, in human resource management, organizations must develop competencies to identify and mitigate biases arising from data collection, model training, and algorithmic deployment. These capabilities are becoming integral to dynamic capabilities that allow firms to adapt effectively to rapidly evolving technological landscapes (Bandara et al., 2025).

The transformative effects of AI are likewise evident in education and professional training. Multiple studies point to a widening gap between the digital and analytical competencies required by the labor market and those perceived among students and recent graduates. This misalignment assumes particular salience in the context of Industry 5.0, which prioritizes human-centric innovation, interdisciplinary collaboration, and ethical technological development (Tiron-Tudor et al., 2025). Bridging this gap necessitates enhanced collaboration among academia, industry, and policymakers to redesign curricula and foster lifelong learning strategies suited to increasingly AI-mediated workplaces.

At the inter-organizational level, AI adoption influences supply chain resilience and ecosystem coordination. Evidence from the Chinese semiconductor industry suggests that digital transformation enhances firms’ ability to manage dynamic risks and adapt to volatile market conditions. However, the interplay among technological adoption, government subsidies, and financial performance appears complex and at times nonlinear, implying that public policies and organizational strategies must be carefully calibrated to prevent unintended consequences (Guo et al., 2025). At the institutional level, reforms in judicial digitalization and regulatory frameworks have been shown to accelerate corporate digital transformation by reducing uncertainty and improving access to financing (Zheng et al., 2025).

The general-purpose character of AI is further exemplified by its diverse applications. In construction, convolutional neural networks have been employed to automate symbol recognition in engineering drawings, substantially reducing analysis time while improving accuracy (Jamieson et al., 2025). In agriculture, deep learning architectures enable high-accuracy detection and classification of crop leaf diseases when combined with advanced segmentation and data augmentation techniques (Ozcan and Polat, 2025). Even within cultural and creative industries, generative AI models such as LoRA are opening novel avenues for the preservation and reinterpretation of intangible cultural heritage through immersive and interactive experiences (Wang and Zhou, 2025). These cases reinforce the view of AI as a foundational technology capable of reshaping a wide array of economic and social sectors.

Despite these advances, several integrative gaps persist in the literature. First, longitudinal evidence linking the strategic deployment of AI to organizational performance and sustainability outcomes remains limited. Second, the institutional and governance conditions required for responsible AI adoption—including ethical frameworks, explainability mechanisms, and data protection standards—continue to be fragmented across studies (Bandara et al., 2025; Erdmann and Toro-Dupouy, 2025). Third, tensions remain between corporate communication strategies concerning AI capabilities and customer expectations regarding trust, transparency, and human interaction (Vorobeva et al., 2025). Finally, empirical findings are still inconclusive concerning the interactions among public policies, financial incentives, innovation ecosystems, organizational agility, and supply chain resilience (Guo et al., 2025; Zheng et al., 2025).

Given this fragmented research landscape, there is an increasing need for integrative analyses that synthesize extant evidence and delineate promising future research directions in the domain of AI and business strategy. Accordingly, the present study seeks to map and interpret the evolution of scientific research on the strategic value of artificial intelligence within global business contexts. To this end, the study combines bibliometric analysis of scientific output with a qualitative synthesis of the field’s most influential contributions. This mixed-methods approach facilitates the identification of dominant thematic clusters, collaboration patterns, and emerging trends, while also enabling deeper interpretation of empirical findings and conceptual advancements.

The contribution of this research is twofold. First, it provides an updated mapping of the scientific landscape concerning AI and business strategy, delineating key thematic axes such as strategic integration of AI in decision-making, data-driven innovation and value creation, ethical governance and explainability, interorganizational cooperation, and sustainability-oriented digital transformation. Second, the study advances an interpretive framework that links heterogeneous empirical results to relevant contextual conditions, including organizational culture, dynamic capabilities, data governance practices, and institutional environments (Bandara et al., 2025; De Felice et al., 2025; Erdmann and Toro-Dupouy, 2025; Festa et al., 2025; Guo et al., 2025; Martin et al., 2025; Mehta et al., 2025; Torrent-Sellens et al., 2025; Wang and Zhou, 2025; Wang Y. et al., 2025).

The remainder of this article is structured as follows. Section 2 describes the methodological design, encompassing the bibliometric procedures and the selection criteria for the qualitative synthesis of influential studies. Section 3 presents the principal results of the bibliometric analysis, including temporal trends, collaboration networks, and thematic co-occurrence patterns. Section 4 discusses these findings in relation to prevailing theoretical perspectives and sectoral contexts, highlighting convergences, contradictions, and emerging research opportunities. Finally, Section 5 summarizes the main conclusions, delineates practical implications for organizations and policymakers, and proposes avenues for future research.

## 2 Methodology

The present study employs a bibliometric review combined with narrative synthesis, rather than a fully structured systematic review. The primary objective is not to exhaustively appraise empirical evidence concerning a specific causal relationship, but rather to map and interpret the intellectual structure and thematic evolution of the literature on the strategic value of artificial intelligence in global business contexts. Accordingly, bibliometric analysis serves as the principal methodological approach, enabling the identification of publication trends, influential authors, collaboration networks, and thematic clusters within the field.

To complement this quantitative mapping, a narrative qualitative synthesis of the most influential publications was undertaken. This interpretive component seeks to elucidate the conceptual contributions and emerging research directions discerned through the bibliometric analysis. The qualitative synthesis thus performs an interpretive rather than evaluative function, providing contextual depth to the patterns revealed by bibliometric indicators, as opposed to conducting a formal evidence synthesis in the manner characteristic of traditional systematic reviews.

While the study does not purport to constitute a complete systematic review, selected elements of transparent literature screening—drawn from PRISMA guidelines—were incorporated to enhance methodological rigor. These include clearly defined search strategies, the selection of core databases (Scopus and Web of Science), duplicate removal, and the application of predetermined inclusion and exclusion criteria. The final qualitative synthesis centers on the 50 most influential articles within the dataset, selected according to citation impact and thematic alignment with the study’s research objectives.

This hybrid approach—bibliometric mapping augmented by narrative synthesis—has been widely adopted in emerging research fields where the aim is to delineate knowledge structures, trace research trajectories, and elucidate conceptual developments, rather than to assess the effectiveness of specific interventions.

### 2.1 Sources of information and search strategy

The collection of scientific data was carried out from the Scopus and Web of Science (WoS) databases, recognized for their international coverage and the quality of their bibliographic records. These platforms were selected for their relevance in management, technology, and innovation studies, as well as for their analytical tools that facilitate the processing and normalization of metadata (Singh et al., 2025; Vasudevan et al., 2025).

The Social Sciences Citation Index (SSCI) and Science Citation Index Expanded (SCI-EXPANDED) collections from WoS were included to ensure the quality and multidisciplinarity of the documents. Regional databases such as SciELO and Google Scholar were not included in the search strategy because they present heterogeneous indexing standards and limited coverage of high-impact international journals in the fields of strategic management and artificial intelligence. The use of Scopus and Web of Science ensures greater consistency in bibliographic metadata and facilitates the application of standardized bibliometric indicators.

The search equations were constructed with Boolean operators and controlled terms that reflect the relationship between artificial intelligence, business strategy, and the global context, applied in the fields of title, abstract, and keywords. The equations used were the following: Scopus:\ TITLE-ABS-KEY(“artificial intelligence” OR “machine learning” OR “deep learning” OR “AI-driven” OR “intelligent systems”) AND TITLE-ABS-KEY(“business strategy” OR “corporate strategy” OR “strategic management” OR “competitive advantage” OR “global business” OR “digital transformation”) Web of Science (WoS): TS = (“artificial intelligence” OR “machine learning” OR “deep learning” OR “AI-driven” OR “intelligent systems”) AND TS = (“business strategy” OR “corporate strategy” OR “strategic management” OR “competitive advantage” OR “global business” OR “digital transformation”) Inclusion and exclusion criteria Selection criteria were applied oriented toward ensuring the thematic relevance and methodological quality of the documents:

#### 2.1.1 Inclusion criteria

- Original articles and peer-reviewed published between 2015 and 2025.
- Publications in English or Spanish with access to full text.

#### 2.1.2 Exclusion criteria

- Non-peer-reviewed works (for example, conferences or preprints).
- Duplicate documents.

After the review of titles and abstracts, studies related to strategic management, technological innovation, digital transformation, and organizational performance that explicitly analyzed the impact of AI on the formulation, implementation, or evaluation of business strategies were included. Additionally, the following were excluded: Purely technical studies on algorithms without a link to the strategic or business realm.

### 2.2 Screening procedure and transparency of the selection process

The screening and data preparation process adhered to transparent reporting principles inspired by the PRISMA guidelines to ensure the reproducibility of the dataset construction. Following the initial searches in Scopus and Web of Science, all retrieved records were exported in compatible bibliographic formats and consolidated into a single unified dataset.

Duplicate records were identified and managed through a two-stage procedure. First, automated duplicate detection was performed during dataset integration, utilizing key bibliographic matching fields (title, authors, and publication year). This was followed by a manual verification step to confirm duplicate matches and to prevent the erroneous exclusion of records exhibiting minor metadata variations.

Title and abstract screening was conducted independently by two reviewers to assess thematic relevance to the study’s scope. Records were evaluated against the following predefined inclusion criteria:

- explicit focus on artificial intelligence or advanced analytics technologies,
- relevance to business strategy, organizational performance, or value creation, and
- publication in peer-reviewed journals indexed in Scopus or Web of Science within the designated time frame.

Disagreements between reviewers during the screening phase were resolved through discussion until consensus was reached, thereby ensuring that the final dataset reflected mutual agreement on thematic relevance and methodological appropriateness.

It should be noted that no further exclusions were applied at the full-text review stage. Given that the bibliometric component of this study seeks to map the overall research landscape of the field rather than to appraise specific empirical findings, all records meeting the inclusion criteria at the title-and-abstract stage were retained in the bibliometric corpus. The subsequent qualitative narrative synthesis was then performed on a purposive subsample comprising the 50 most influential articles, selected on the basis of citation impact and conceptual alignment with the research objectives.

### 2.3 Data extraction and cleaning procedure

Records retrieved from Web of Science and Scopus were exported in .xls and .csv formats, respectively, and subsequently integrated into a unified dataset using R (version 4.4.2) (der Van Elst, 2024). As an initial harmonization step, article titles were standardized by converting them to lowercase across both databases to facilitate duplicate detection. Duplicate records between Web of Science and Scopus were identified through title-matching procedures employing dplyr joins and subsequently classified into three categories: records exclusive to Scopus, records exclusive to Web of Science, and records indexed in both databases.

Following deduplication, the unique records and the shared (overlapping) records were merged to form a single analytical corpus. Bibliographic fields from both sources were then standardized to ensure structural comparability, encompassing authors, title, publication year, source title, citation counts, DOI, keywords, references, affiliations, and abstracts.

The resulting dataset underwent keyword-based filtering applied to titles, abstracts, and author keywords. In the first filtering stage, only records containing at least one artificial intelligence-related term—such as artificial intelligence, machine learning, deep learning, AI-driven, or intelligent systems—were retained. In the second stage, the dataset was further restricted to those records also containing at least one business-related term, including business strategy, corporate strategy, strategic management, competitive advantage, global business, or digital transformation.

To enhance thematic precision, bibliometric studies and systematic literature reviews were excluded from the final analytical corpus. The cleaned dataset was then exported and served as the foundation for the subsequent bibliometric analysis and qualitative narrative synthesis.

Productivity, measured as the number of publications, and impact, evaluated through citations received, were synthesized by year and represented graphically using cumulative area charts and bar charts, which allowed visualizing emerging trends in the field. Additionally, the most influential journals on the topic were analyzed.

Additionally, VOSviewer software (version 1.6.20) was used to generate keyword co-occurrence maps. These visualizations provided a graphical representation of the structure and interconnections within the research field on AI-driven strategic value in global businesses.

Initially, 13,614 records were identified from two databases: Scopus (11,235) and Web of Science (2,379). After applying the inclusion and exclusion criteria, 4,528 records remained (2,571 in Scopus and 1,957 in WoS). During screening, 931 duplicates were removed, and studies were discarded for not meeting the objectives after title and abstract review (927). Finally, 2,670 documents were evaluated for eligibility, and none were excluded, constituting the final corpus of the analysis.

The structure of the bibliometric networks allowed identifying thematic clusters related to AI adoption, decision automation, algorithmic ethics, competitive advantage, and organizational transformation.

### 2.4 Qualitative analysis

To complement the bibliometric mapping with a deeper interpretive perspective, a qualitative narrative synthesis of the most influential studies was undertaken. The selection of the final 50 articles was guided by a purposive, relevance-based sampling strategy commonly employed in mixed-method bibliometric reviews. Specifically, articles were ranked according to three primary criteria: citation impact within the dataset, thematic relevance to the core research topic, and their conceptual contribution to understanding the strategic deployment and value creation potential of artificial intelligence in business contexts.

The decision to focus on the 50 most influential studies enables the analysis to capture the central intellectual structure of the field while preserving sufficient analytical depth and interpretive richness. This sample size aligns with established practice in prior hybrid bibliometric–qualitative studies, where a deliberately focused subset of highly cited or conceptually pivotal works is examined in detail to elucidate dominant thematic clusters, prevailing theoretical frameworks, and emergent research trajectories. This review focused on examining the conceptual and empirical approaches used to address the strategic value driven by AI in global companies. The articles were categorized according to three analytical dimensions:

1. Strategic dimension: how AI redefines the planning, formulation, and execution of business strategies.
2. Technological dimension: use of algorithms, machine learning, and predictive analysis for decision-making.
3. Organizational dimension: impact of AI on the structure, the culture, and corporate leadership.

This procedure allowed synthesizing the main theoretical and practical contributions of the most influential literature, as well as detecting research gaps, emerging trends, and areas of opportunity for the future development of the field.

## 3 Results

**Figure 1:**
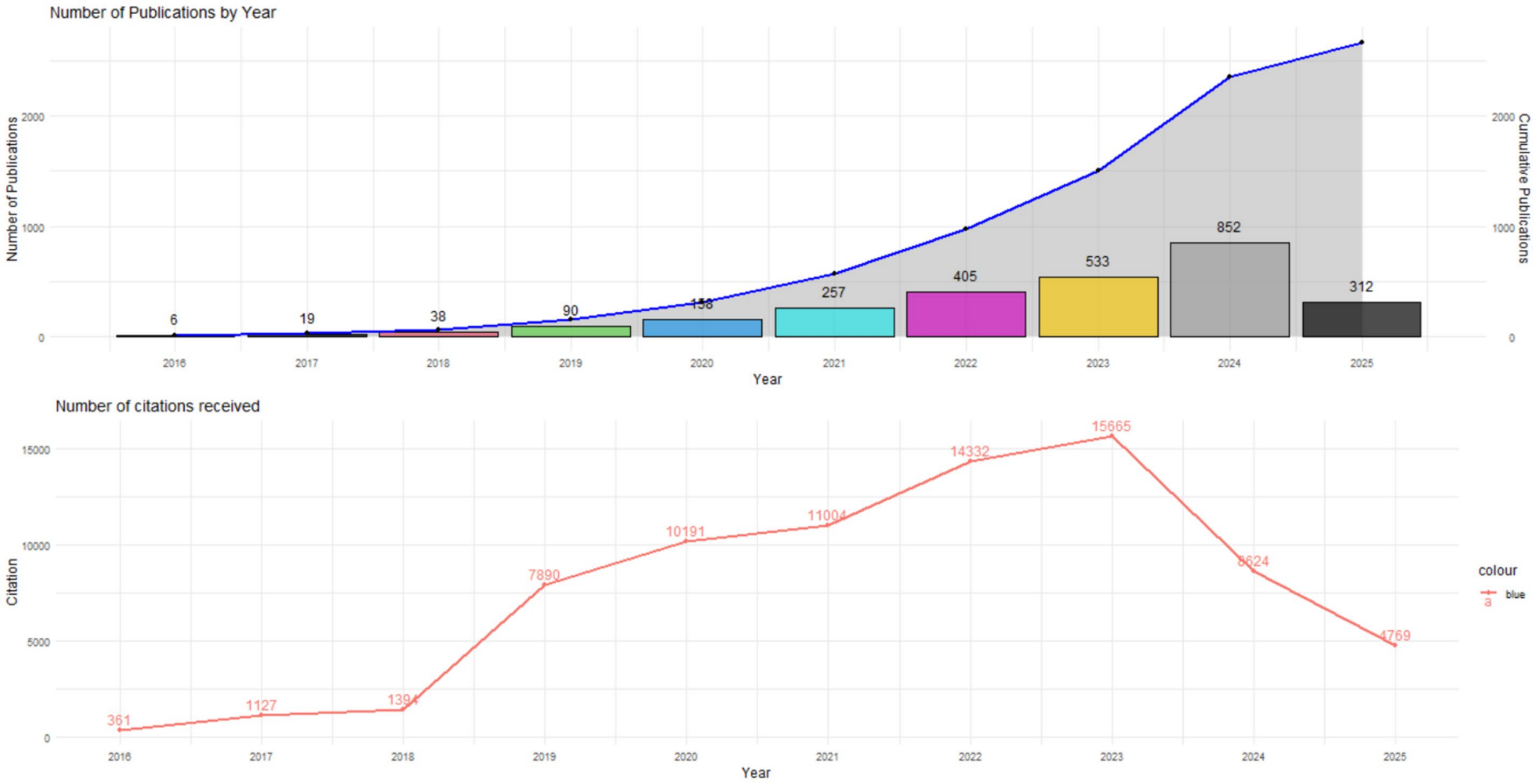
Evolution of scientific publications and citation trends on AI-driven strategic value in global businesses (2016–2025).

Analysis of Productivity and Citations Figure 1 presents the evolution of scientific production and the number of accumulated citations in the field of strategic value driven by artificial intelligence (AI) in global businesses, during the period 2016–2025. The results reflect an exponential growth in productivity and a nonlinear trend in citation impact, which evidences both the consolidation of the field and its thematic diversification in the most recent years.

### 3.1 Initial exploration phase (2016–2018)

In this first stage, scientific production was incipient and characterized by its conceptual and theoretical orientation. A total of 63 publications and 2,882 accumulated citations were recorded, concentrated in pioneering studies on the application of AI in strategic decision-making.

- In 2016, only 6 articles addressed the topic, with 361 citations, centered mainly on the automation of processes and predictive analytics as emerging sources of competitive advantage.
- During 2017, productivity increased to 19 documents, reaching 1,127 citations, with research that began to explore the integration of AI in organizational management and business learning.
- In 2018, production rose to 38 articles with 1,394 citations, marking the beginning of sustained interest in AI as a driver of innovation in corporate environments.

In this phase, publications were predominantly oriented toward theoretical management models and exploratory studies, with limited international cooperation. Universities in the United States and the United Kingdom led the discussion, while contributions from Asia and Latin America were still marginal.

### 3.2 Accelerated growth phase (2019–2022)

The period between 2019 and 2022 represents the phase of expansion and academic consolidation of the field. Scientific production grew exponentially, going from 90 publications in 2019 to 405 in 2022, which equates to a 350% increase in 4 years. Together, these years accumulate 44,417 citations, confirming the maturity and relevance of the theme within the literature on business management and digital transformation.

- In 2019, 7,890 citations were counted distributed among 90 documents, which evidences the emergence of a robust theoretical body on AI as a source of dynamic competitive advantage.
- In 2020, production increased to 158 articles, with 10,191 citations, driven by the context of the COVID-19 pandemic, which stimulated research on organizational resilience, operational efficiency, and digital adaptation.
- In 2021, with 257 publications and 11,004 citations, AI consolidated itself as a pillar of data-based corporate strategy, integrating predictive analysis, machine learning, and decision automation.
- Finally, 2022 reached the peak of impact with 405 publications and 14,332 citations, marking a turning point toward more interdisciplinary approaches that combine strategic management, algorithmic ethics, and business sustainability.

During this stage, scientific production showed sustained growth both in quantity and quality. Multicentric empirical studies and systematic reviews were published, with a growing presence in high-impact journals (Q1 and Q2). International co-authorship intensified, configuring collaboration networks between institutions in North America, Europe, and Asia.

### 3.3 Consolidation and diversification phase (2023–2025)

Between 2023 and 2025, the literature experiences a phase of scientific maturity and thematic diversification. Although productivity continued to increase, the total number of citations showed a downward trend, possibly due to field saturation and the shift of interest toward emerging subthemes such as generative AI, data governance, and algorithm ethics.

- In 2023, the historical maximum of citations (15,665) and 533 publications were recorded, consolidating AI as a transversal strategic axis for global competitiveness.
- In 2024, productivity reached 852 articles, but the total citations fell to 8,624, which could be interpreted as a process of fragmentation of research lines or a redistribution of impact toward new thematic domains.
- For 2025, with 312 publications and citation data of 4,769, a relative stabilization in production is observed, associated with the rise of applied research on generative AI, business intelligence, and technological sustainability.

This stage is distinguished by the emergence of studies focused on the strategic value of explainable AI (XAI), corporate ethics of algorithms, and the synergy between automation and human decision-making. However, the decline in the number of recent citations suggests that the newest articles still require time to consolidate their visibility and achieve recognition within the international academic circuit.

### 3.4 Most influential scientific journals

Table 1 shows the distribution of publications and citations from the main scientific journals that have addressed the study topic. A marked concentration of productivity in high-impact journals (Q1) is observed, which evidences the sustained interest of the international scientific community in this line of research and its positioning within consolidated publications in the field of sustainability, technological innovation, and business management.

**Table 1:** Most influential scientific journals in the field of AI and business strategy.

Source title	Cantidad	Citas	Quartil
Sustainability (Switzerland)	93	3,268	Q1
IEEE Access	35	1,169	Q1
Technological Forecasting and Social Change	27	2,134	Q1
Applied Sciences (Switzerland)	22	445	Q2
Electronics (Switzerland)	21	277	Q2
Sensors	17	341	Q1
Journal of Ecohumanism	15	28	Q2
Journal of Business Research	14	1,274	Q1
Energies	14	279	Q1
Computers and Industrial Engineering	12	414	Q1

First, the journal Sustainability (Switzerland) leads widely in the number of publications (93) and in the total number of citations (3,268), which positions it as the main channel for disseminating knowledge in this field. Its Q1 quartile reflects a high level of visibility and impact, in line with its multidisciplinary focus on sustainability, innovation, and management.

Second, IEEE Access (35 articles, 1,169 citations) and Technological Forecasting and Social Change (27 articles, 2,134 citations) stand out for their high average citation per article, which suggests that their contributions are especially influential. In particular, Technological Forecasting and Social Change presents a citation/article ratio greater than 79, indicating a solid international projection and the relevance of its contributions on topics of digital transformation and technological foresight.

In the Q2 quartile range, journals such as Applied Sciences (Switzerland; 22 articles, 445 citations) and Electronics (Switzerland; 21 articles, 277 citations) are located, which maintain considerable productivity but with medium impact compared to Q1 journals. This reflects the thematic diversification and the opening of intermediate-level publication spaces within the same research field.

On the other hand, the journal Journal of Ecohumanism (15 articles, 28 citations, Q2) constitutes a particular case, given its low citation index in relation to its productivity. This suggests that its more recent and philosophical approach is still in the process of consolidation in the scientific community.

Finally, journals such as Journal of Business Research (14 articles, 1,274 citations) and Computers and Industrial Engineering (12 articles, 414 citations), both in Q1 quartile, stand out for combining moderate productivity with high impact per article, which indicates that the studies published in these journals generate strong influence in the literature on artificial intelligence, business sustainability, and digital transformation.

Overall, the evidence suggests that the analyzed research is concentrated in Q1 journals, which reinforces the scientific solidity of the field and its multidisciplinary character, with emphasis on sustainability, engineering, management, and technological innovation. The diversity of Q1 and Q2 journals reflects both the maturity of the topic and its expansion toward applied and frontier areas.

### 3.5 Collaboration between institutions

**Figure 2:**
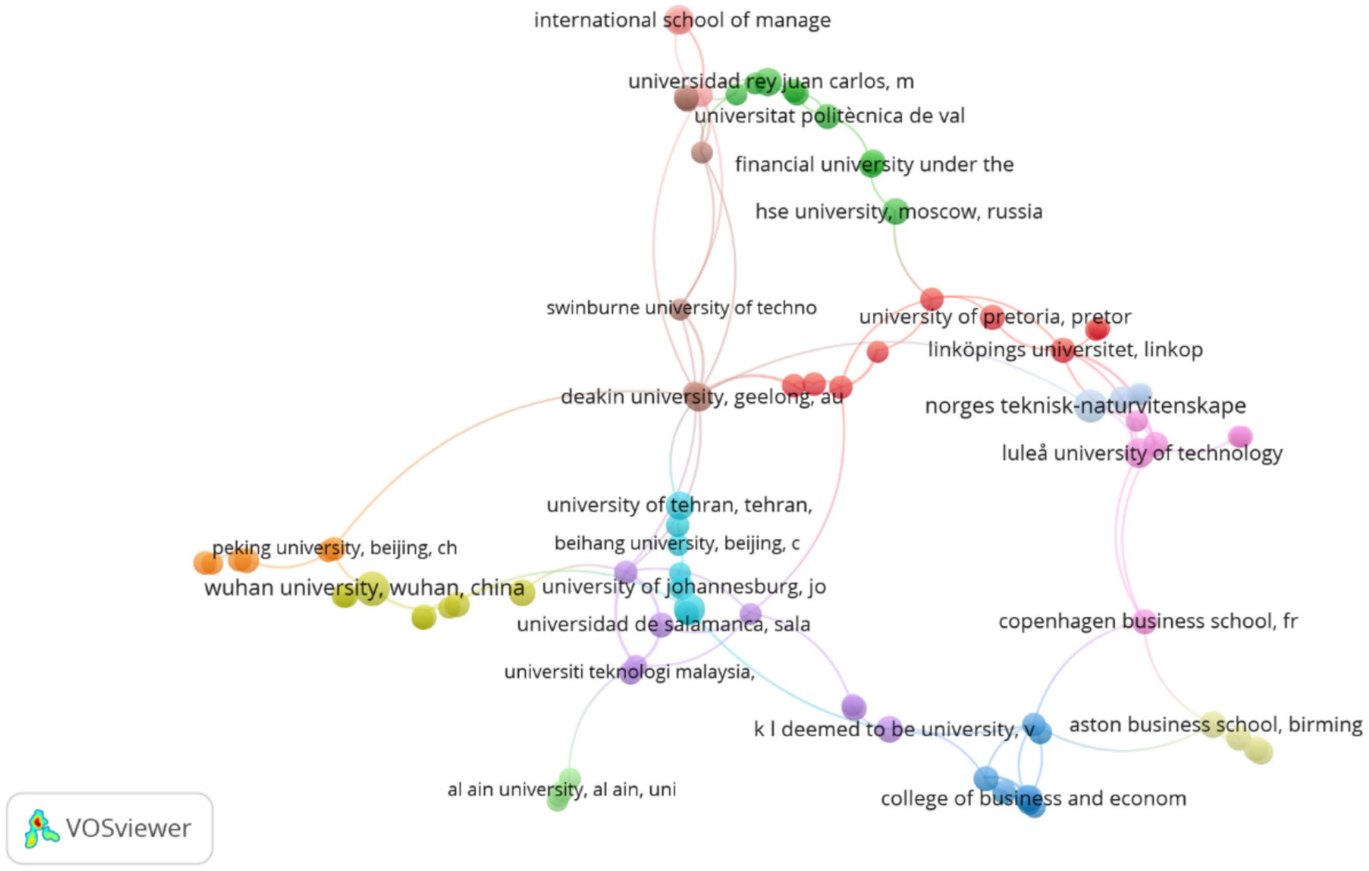
Institutional co-authorship network on AI-driven strategic value in global businesses (2016–2025).

Figure 2 represents the institutional co-authorship map generated with VOSviewer, in which the main collaboration networks between universities and research centers that publish on the analyzed topic are visualized. The nodes indicate institutions, while the size reflects productivity (number of documents) and the thickness of the links expresses the total link strength, that is, the intensity of inter-institutional collaboration. Most Influential Institutional Cores According to the table, several universities stand out with a notable combination of high productivity, high number of citations, and strong international linkage. Among them, the following stand out:

- Luleå University of Technology (Sweden), with 10 documents, 768 citations, and a total link strength of 10, consolidating itself as a Nordic reference center with direct connections to European and Australian institutions.
- Deakin University (Australia), with 9 documents, 230 citations, and 11 link strength, which reflects its central role as an articulator between networks in Europe, Asia, and Oceania.
- University of Pretoria (South Africa; 7 documents, 448 citations) and Copenhagen Business School (Denmark; 7 documents, 1,254 citations) form a relevant axis of academic collaboration between the global north and south, especially in studies on sustainability, innovation, and organizational development.

Institutions with High Visibility and Thematic Specialization Several European universities present high levels of citation in relation to their productivity:

- Aston Business School (United Kingdom) records 8 articles and 1,320 citations, evidencing high academic visibility and leadership in lines of research applied to business management and organizational intelligence.
- Technische Universität Berlin (1,073 citations) and ETH Zürich (777 citations) stand out for their methodological and technical contribution, especially in quantitative analysis and technological innovation.
- University of Tehran (Iran) and Indian Institute of Technology Kharagpur (India) stand out for their growing intervention in Asian networks, with a significant level of citations (more than 780 each), which reflects their influence in the Euro-Asian axis.

### 3.6 Formation of collaboration communities

The map evidences groupings by regions or thematic affinities:

- The European cluster (in green) integrates Spanish universities (Universidad Rey Juan Carlos, Universitat Politècnica de València, Universidad de Salamanca), along with institutions from the United Kingdom and Nordic countries, with strong connections toward Central Asia and the Middle East.
- The Asian cluster (in orange) mainly includes Chinese universities (Wuhan University, Peking University, Beihang University), with a concentrated collaboration pattern and lower interconnectivity with Western institutions.
- The oceanic and African cluster (in purple and light blue) links Deakin University, University of Pretoria, and Swinburne University of Technology, evidencing south–south cooperation and contributions on topics of digitalization and sustainability.

### 3.7 Emerging networks and peripheral actors

Institutions such as the University of Johannesburg (South Africa), Universiti Teknologi Malaysia, and the University of La Rioja (Spain) present intermediate levels of collaboration (link strength between 2 and 4), which suggests an emerging participation in the global network.

Likewise, centers such as the International School of Management (Germany) and the Financial University under the Government of the Russian Federation (Russia) reinforce the interdisciplinary component of the network. Figure 2 demonstrates a polycentric structure of scientific cooperation, where Europe (particularly Scandinavia and the United Kingdom) and Asia (China and India) lead academic production, while Latin America and Africa play a growing role in specific alliances. The general pattern reflects the maturity and globalization of the research field, with a trend toward the integration of transcontinental networks and the consolidation of intermediate nodes that articulate knowledge between regions.

### 3.8 Leading countries in scientific production

**Figure 3:**
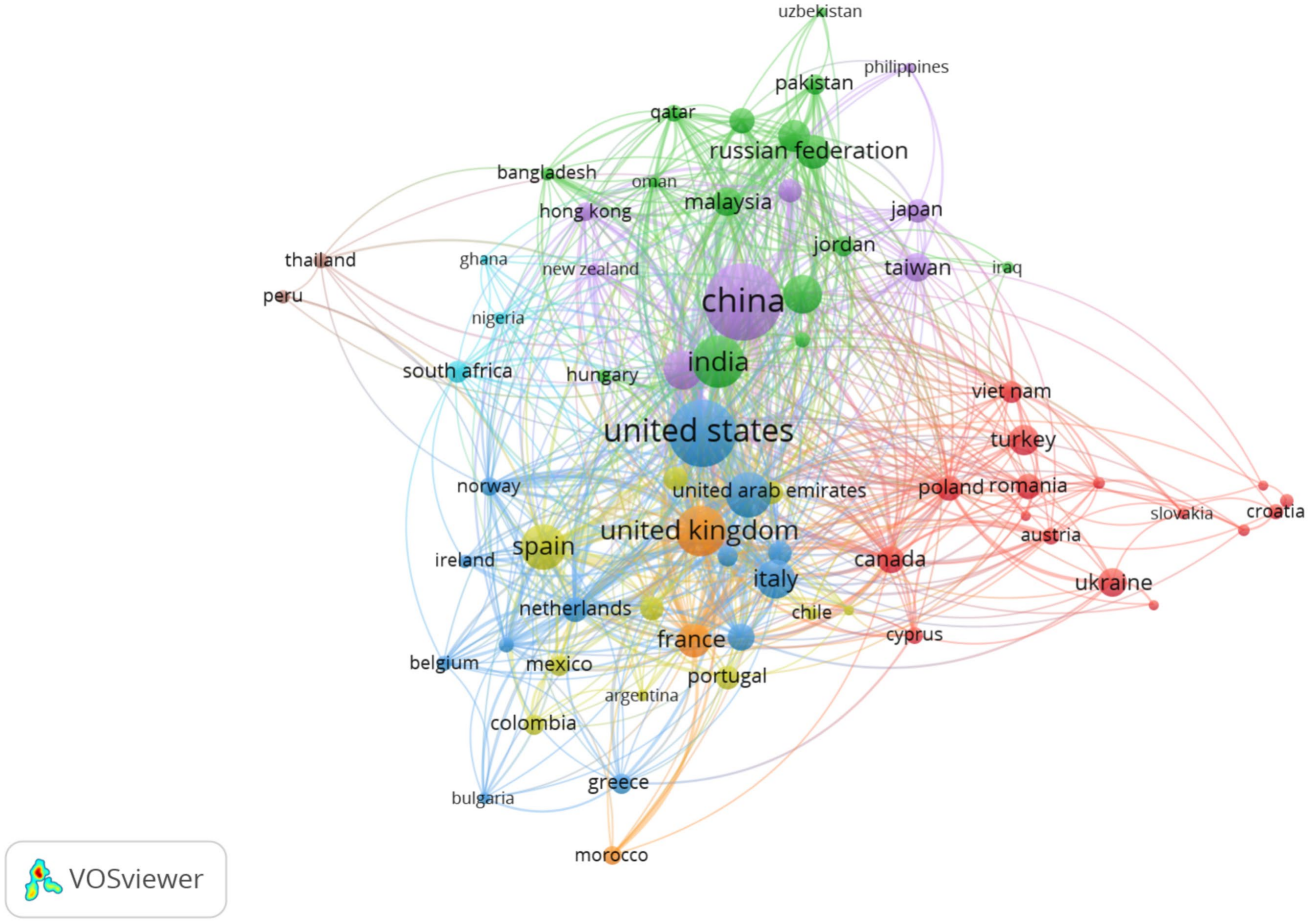
International country co-authorship network on AI-driven strategic value in global businesses (2016–2025).

Figure 3 represents the international collaboration map between countries generated with VOSviewer, based on the co-authorship of academic publications. The size of the nodes reflects the number of documents produced by each country, while the intensity of the links (lines) and their thickness indicate the degree of scientific collaboration (total link strength). The colors correspond to the different geographic and thematic clusters, which allow visualizing the formation of interconnected investigative communities at the global level.

### 3.9 Global core of production and influence

The results show a highly dense and polycentric network, led by three academic powers: China [389 documents, 8,078 citations, Total Link Strength (TLS) = 215], United States (288, 14,357, TLS = 245) and United Kingdom (167, 14,933, TLS = 252). These three nations configure the central core of the international scientific system, standing out both for their volume of production and for their influence in terms of citation and interconnectivity.

In this core, a bidirectional flow of cooperation is observed, especially between the United States–United Kingdom–China axis, which articulates the knowledge transfer around artificial intelligence, machine learning, and digital transformation. Their central position within the graph indicates an epistemic leadership capacity, by serving as intermediaries in the global circulation of ideas, methodologies, and theoretical frameworks.

### 3.10 European cluster: integration and high collaborative density

The European cluster is distinguished by a wide interconnection between countries of the European Union and the European Economic Area. Germany (140; 8,888; TLS = 130), France (76; 7,199; TLS = 142), Italy (97; 6,209; TLS = 104), Spain (138; 6,304; TLS = 120) and Netherlands (42; 3,985; TLS = 103) stand out, which form a block of high productivity and mutual collaboration.

This network suggests the existence of transnational research consortia, funded by European programs such as Horizon Europe, oriented toward innovation, sustainability, and digitalization projects. Likewise, Nordic countries such as Sweden, Norway, and Finland complement this network, contributing with high-impact studies on technological governance and AI ethics.

### 3.11 Asian cluster: expansion and technological cooperation

The Asian cluster groups India (180; 10,356; TLS = 172), Japan (37; 1,281; TLS = 28), South Korea (95; 2,700; TLS = 44), Singapore (36; 4,113; TLS = 69), Malaysia (54; 1,270; TLS = 74) and Indonesia (42; 415; TLS = 34). This block reflects a progressive expansion of technological research on the continent, characterized by an applied orientation in industrial, educational, and digital health sectors.

The strong connection between India and China, and their links with the United Kingdom and United States, reveal a dynamic South–North collaboration, centered on technology transfer and the development of artificial intelligence algorithms adapted to local contexts.

### 3.12 Latin American cluster and emerging economies

The Latin American cluster is composed of Mexico (29; 563; TLS = 25), Brazil (37; 936; TLS = 35), Colombia (28; 632; TLS = 17), Chile (12; 148; TLS = 15) and Argentina (9; 92; TLS = 10). This group maintains relevant connections with Spain and the United States, evidencing collaborative dependence toward consolidated research centers.

Although the density of their links is lower, the growing number of publications suggests a strengthening of regional capacities and a progressive insertion into the international scientific network. Additionally, the participation of emerging countries from the Middle East is observed, such as Saudi Arabia (71; 4,518; TLS = 105), United Arab Emirates (39; 1,340; TLS = 48) and Qatar (20; 3,768; TLS = 48), which position themselves as strategic nodes of interregional cooperation.

### 3.13 Peripheral collaborations and emerging actors

In the periphery of the map are located countries with lower production volume, but with significant links toward the central nodes. Among them, Denmark (17; 4,452; TLS = 56), Switzerland (39; 4,523; TLS = 76) and Canada (51; 2069; TLS = 64) stand out, which maintain collaborations of high scientific quality with the global leaders.

The appearance of countries such as Bangladesh, Pakistan, Egypt or Ghana within the network reflects an incipient process of internationalization, favored by cooperation with Asian and European powers.

### 3.14 Thematic area

**Figure 4:**
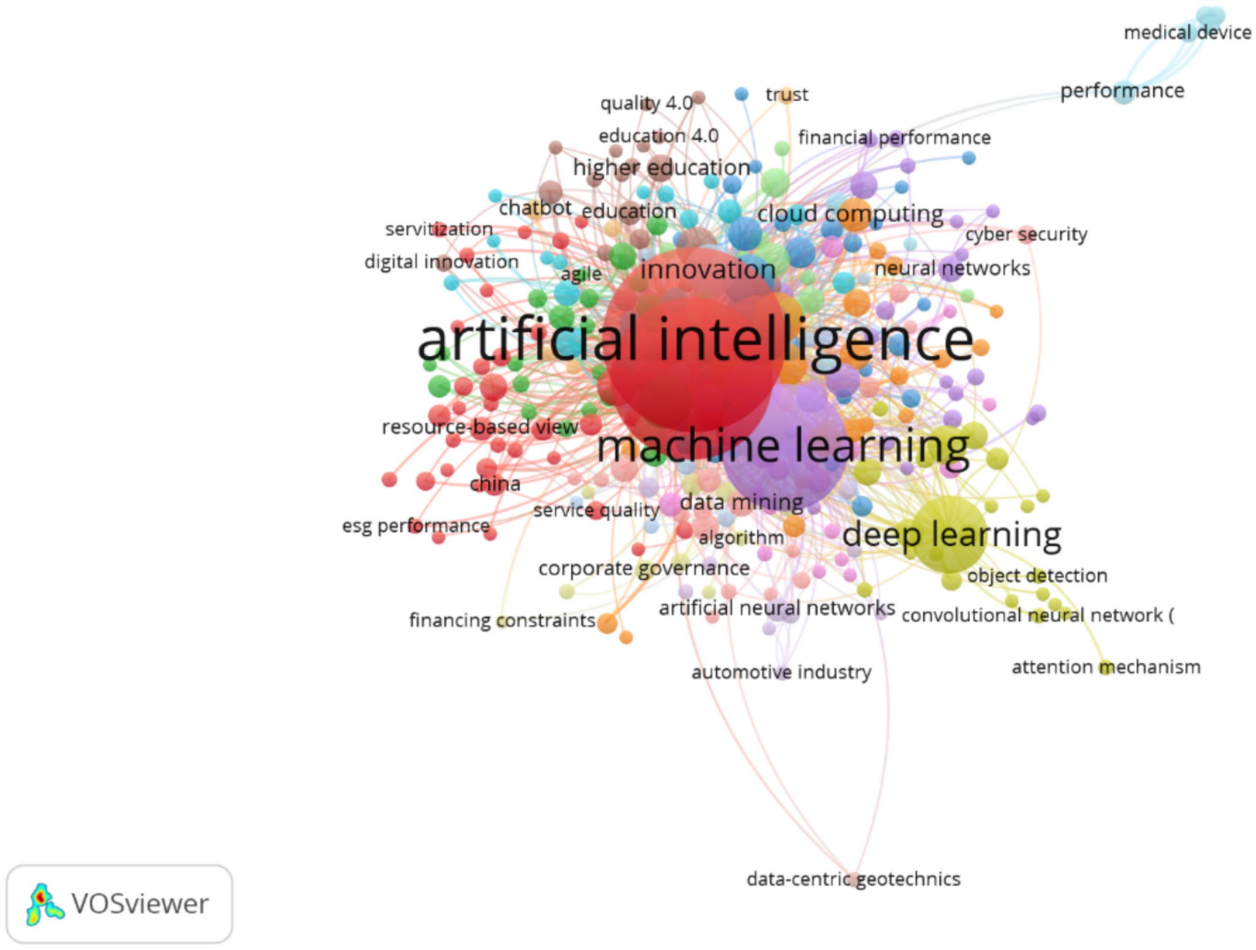
Keyword co-occurrence network on AI-driven strategic value in global businesses (2016–2025).

Figure 4 represents the term co-occurrence map elaborated with VOSviewer, in which the nodes indicate keywords and the links reflect the frequency with which these appear jointly in the analyzed articles. The size of the nodes corresponds to the number of occurrences, while the color distinguishes the thematic clusters. This type of visualization allows identifying the conceptual cores and emerging trends around the strategic value of artificial intelligence (AI) in global businesses.

### 3.15 Central core: artificial intelligence and machine learning

The terms “artificial intelligence” (841 occurrences, link strength 1754) and “machine learning” (407 occurrences, link strength 798) constitute the densest conceptual core of the network, evidencing their transversal and integrative role in the literature. Both concepts are closely linked to notions such as “deep learning” (151; 226), “data mining” (19; 34) and “neural networks” (17; 36), forming a technological cluster centered on the development of algorithms, predictive models, and deep learning.

This convergence reflects a transition of AI from a purely technical approach toward applications oriented to organizational value, where analytical and predictive capacity becomes a source of competitive advantage.

### 3.16 Cluster of digitalization and organizational transformation

The second thematic group revolves around “digital transformation” (661 occurrences, 1,370 link), closely related to “innovation” (69; 174), “industry 4.0” (118; 321), “digitalization” (67; 177) and “blockchain” (68; 234).

These terms configure a cluster of technological and strategic modernization, where AI is associated with automation processes, digital servitization, and corporate sustainability strategies. Concepts such as “cloud computing” (31; 105) and “cybersecurity” (31; 85) evidence the focus toward secure and scalable digital infrastructures.

The density of this group suggests that AI acts as a catalyst for digital innovation, transforming business models, value chains, and productive processes in various industrial sectors.

### 3.17 Cluster of management, value, and business performance

Another relevant focus is formed by terms such as “competitive advantage” (42; 97), “strategic management” (28; 70), “performance” (16; 48), “corporate governance” (12; 24) and “firm performance” (13; 24).

This set of keywords constitutes the strategic and economic axis of the map, in which it is analyzed how the adoption of AI drives efficiency, organizational agility, and value creation. The presence of terms such as “digital technologies” (45; 136) and “business model innovation” (5; 12) reinforces the idea that AI is perceived as a strategic resource that reconfigures competitive advantages and favors data-based decision-making.

### 3.18 Cluster of applied innovation and sustainability

An emerging group, associated with the brown color in the figure, is composed of terms such as “sustainability” (52; 163), “resource-based view” (15; 32), “esg performance” (6; 12) and “green innovation” (10; 13).

This cluster represents the sustainable dimension of the strategic value of AI, where digital innovation models are linked to corporate social responsibility, energy efficiency, and sustainable development goals (SDGs). The interaction between AI, green innovation, and responsible management indicates a conceptual evolution toward more ethical and sustainable business models.

### 3.19 Emerging academic and technological cluster

Finally, terms such as “ChatGPT” (19; 49), “large language models” (9; 24), “generative AI” (18; 45) and “explainable artificial intelligence” (7; 16) stand out. These form an emerging cluster linked to recent advances in generative models and to debates on transparency and ethics in AI. Their recent appearance suggests a new research frontier, centered on interpretability, algorithmic biases, and the social implications of AI in strategic decision-making.

### 3.20 Interpretive synthesis

Figure 4 evidences a polycentric structure organized around five major axes: (1) the technical core of AI and machine learning, (2) digital transformation and Industry 4.0, (3) strategic management and business value creation, (4) sustainability and responsible innovation, and (5) emerging generative models.

This distribution suggests that research on strategic value driven by AI has evolved from initial technological approaches toward more multidimensional perspectives oriented to organizational and social impact. The interaction between clusters reflects a growing integration between technology, strategy, and sustainability, consolidating AI as a central axis of contemporary global competitiveness.

## 4 Qualitative analysis

### 4.1 Descriptive summary of the studies

Table 2 summarizes the main thematic patterns identified in the qualitative analysis of the 50 most influential studies. The detailed comparison of individual studies was moved to the supplementary material (Appendix A) to improve readability and focus the discussion on the most relevant analytical patterns.

**Table 2:** Key thematic patterns identified in the qualitative review of the 50 most influential studies.

Analytical dimension	Dominant pattern	Supporting studies (examples)
AI integration into strategy	Most studies highlight AI as a strategic tool for predictive analytics, decision support, and strategic planning.	Dogru et al. (2025); Meinhold et al. (2025); Xue et al. (2025); Khan et al. (2025)
Innovation and value creation	AI is widely associated with product and service innovation, data-driven business models, and operational efficiency.	Li Y. et al. (2025); Zhou et al. (2025); Wang and Zhang, 2025a; Parra-López et al. (2025)
Ethical and governance challenges	Several studies emphasize issues such as privacy, algorithmic bias, transparency, and regulatory compliance.	Dogru et al. (2025); Chen and Zhang (2024); Saura and Bužinskienė (2025); Fosso Wamba et al. (2025)
Strategic alliances and ecosystems	Some studies highlight the importance of interorganizational collaboration and digital ecosystems to scale AI adoption.	Xue et al. (2025); Lokanan and Maddhesia (2025); Oulefki et al. (2025)
Startups vs. established firms	Startups tend to emphasize experimentation and agility, while established firms focus on scaling and integration of AI capabilities.	Almansour (2024); Fosso Wamba et al. (2025); Zhou et al. (2025)

The geographic focus is global, with contributions from technologically advanced economies and emerging markets, which reflects interdisciplinary perspectives between the domains of business, technology, and international relations. Together, this comparison elucidates how AI reconfigures strategic decision-making, innovation ecosystems, ethical governance, alliance dynamics, and differential strategies between startups and established companies, directly addressing the central research questions and indicating the need for robust empirical validations, longitudinal designs, and comparative intersectoral and international analyses.

A detailed comparison of all reviewed studies is provided in Appendix A.

### 4.2 Critical analysis and synthesis

The reviewed literature from the 50 most cited studies evidences the transformative role of artificial intelligence (AI) in the configuration of business strategy, value creation, and competitive advantage in global contexts. The findings coincide in that the integration of AI in strategic decision-making enhances operational efficiency, organizational agility, and innovation capacity, reinforcing its articulation with consolidated management frameworks such as resource and capabilities theory and dynamic capabilities (Table 3).

**Table 3:** Strengths and weaknesses: thematic analysis of AI’s role in strategic management and global business (2016–2025).

Aspect	Strengths	Weaknesses
AI in strategic decision-making	The literature shows that AI strengthens planning and strategic decision-making through predictive analytics, machine learning, and scenario simulation, improving accuracy, agility, and risk mitigation in uncertain contexts. Empirical studies and sectoral applications demonstrate integration with classical management frameworks—such as dynamic capabilities and resource-based views—theoretically anchoring its contribution to competitive advantage (Dogru et al., 2025; Li H. et al., 2025; Naeem et al., 2025; Secundo et al., 2025).	Limitations persist regarding data quality and governance, socio-technical integration complexity, and organizational readiness. A portion of the evidence favors short-term operational improvements over sustained strategic effects, and longitudinal impact evaluations remain scarce (Dogru et al., 2025; Li H. et al., 2025; Naeem et al., 2025; Secundo et al., 2025).
Value creation and competitive advantage driven by AI	Findings converge on AI’s role in catalyzing product/service innovation, operational efficiency, and personalized experiences, shaping new data-intensive business models and customer-oriented ecosystems. AI also plays a role in orchestrating dynamic capabilities that sustain competitive advantage (Dogru et al., 2025; Li H. et al., 2025; Naeem et al., 2025; Secundo et al., 2025).	Fine distinctions between startups and incumbents in value capture remain underrepresented, especially in contexts of resource constraints, organizational inertia, and dependency on platform providers. Ethical and governance risks related to value creation (bias, privacy, explainability) still lack systematic empirical treatment (Almansour, 2024; Carayannis et al., 2025; Dogru et al., 2025; Li Y. et al., 2025).
Ethical, governance, and implementation challenges	A significant portion of the literature addresses algorithmic bias, privacy, and transparency, proposing responsible governance frameworks—including explainable AI (XAI)—aligned with corporate ethics and risk management (Dogru et al., 2025; Li H. et al., 2025; Varriale et al., 2025; Meinhold et al., 2025).	Conceptual or narrative analyses with limited validation predominate; there is a lack of comparative studies assessing the effectiveness of governance practices across sectors and jurisdictions. Cross-border coordination in international alliances and global supply chains is scarcely explored from a regulatory perspective (Dogru et al., 2025; Li H. et al., 2025; Secundo et al., 2025; Liu et al., 2025).
AI in international relations and strategic alliances	The strategic role of AI in reshaping alliances, platforms, and global ecosystems is recognized, facilitating cross-border coordination, interorganizational learning, and synergy capture in international value chains (Secundo et al., 2025; Liu et al., 2025; Chen and Zhang, 2024; Zhou et al., 2025).	Evidence is incipient and fragmented; comprehensive models integrating technological, political, and cultural dimensions are still lacking. Regulatory heterogeneity and divergent ethical standards hinder alliance scalability and data interoperability (Secundo et al., 2025; Liu et al., 2025; Dogru et al., 2025).
Adoption of AI in startups vs. established firms	Studies describe how startups use AI to explore niches, accelerate learning, and leverage alliances with technology platforms, while established firms orchestrate AI to scale, standardize, and capture data-based economies of scope (Almansour, 2024; Carayannis et al., 2025; Khan et al., 2025; Muhammad et al., 2025).	There is a lack of rigorous comparative analyses on differential outcomes (performance, time-to-market, resilience), and more evidence is needed on strategic trade-offs under financing and complementary capability constraints (Almansour, 2024; Carayannis et al., 2025; Khan et al., 2025).
Methodological approaches and research robustness	Methodological diversity—bibliometrics, case studies, surveys, and empirical modeling—offers a multifaceted understanding of the phenomenon and enriches theoretical and practical triangulation (Naeem et al., 2025; Secundo et al., 2025; Chen and Zhang, 2024; Almansour, 2024).	Method heterogeneity limits comparability of findings; dependence on secondary data and small samples persists, with few longitudinal or large-scale studies assessing long-term strategic effects (Naeem et al., 2025; Secundo et al., 2025; Chen and Zhang, 2024
Frameworks for AI integration into strategy	Frameworks are proposed linking AI technologies with strategic planning, dynamic capabilities, organizational learning, and governance—including human-AI collaboration and explainability as design principles (Dogru et al., 2025; Naeem et al., 2025; Meinhold et al., 2025; Secundo et al., 2025)	Implementation gaps persist: resistance to change, skill deficits, and limited empirical validation of frameworks; in addition, many models fail to capture sectoral variations or institutional differences between countries (Dogru et al., 2025; Naeem et al., 2025; Meinhold et al., 2025).

Among the main strengths, the consolidation of an interdisciplinary theoretical body that connects AI with strategic management, sustainability, and corporate governance stands out, supported by empirical, bibliometric, and case studies. Likewise, methodological diversity allows capturing different dimensions of the phenomenon, expanding the understanding of its impact on productivity, innovation, and international competitiveness.

However, notable weaknesses persist. The investigations reveal gaps in the practical operationalization of AI in complex organizational contexts, as well as limitations around ethical governance, data reliability, and institutional preparedness. The comparative evidence between startups and established companies continues to be limited, with scant analysis on the differential effects of AI adoption according to resources and structures.

Overall, the scientific landscape describes an evolving ecosystem of strategic transformation, where AI acts as a catalyst for change but also as a source of new challenges. It is recommended to advance toward longitudinal, multiscale research with robust empirical validation that delves into the relationship between AI, strategy, and organizational sustainability, ensuring a responsible and equitable implementation of its transformative potential.

### 4.3 Thematic literature review

The thematic analysis derived from the 50 most cited articles reveals eight dominant lines of research (Table 4). First, AI applied to corporate strategy and decision-making concentrates the greatest attention, with studies that explore the use of predictive analytics and machine learning to optimize resource, risk, and performance management. Second, digital innovation driven by AI appears as a driver of competitive advantage, highlighting its role in servitization, personalization, and value ecosystems.

**Table 4:** Dominant research themes in AI-driven strategic value: prevalence and descriptions from the 50 most influential articles (2016–2025).

Theme	Appears in	Theme description
AI in strategic decision-making and operational efficiency	32/50 Articles	The literature documents that AI increases the accuracy, speed, and quality of strategic decision-making through predictive analytics, machine learning, and optimization, enabling planning under uncertainty and performance improvements at corporate and sectoral levels (Dogru et al., 2025; Li H. et al., 2025; Naeem et al., 2025). These mechanisms facilitate risk mitigation and resource orchestration, with evidence across services, manufacturing, and sustainability domains (Meinhold et al., 2025; Secundo et al., 2025).
AI-driven innovation and competitive advantage	41/50 Articles	AI operates as an innovation catalyst by enabling new products/services, digital ecosystems, and data-based differentiation; it also reinforces dynamic capabilities and value capture in highly competitive contexts (Dogru et al., 2025; Li H. et al., 2025; Naeem et al., 2025). The field highlights effects on performance and strategic positioning, as well as the articulation of AI with processes of opportunity discovery and exploitation (Secundo et al., 2025; Liu et al., 2025).
Strategic alliances and partnerships in AI contexts	11/50 Articles	Interorganizational alliances and technological partnerships emerge as critical pathways for scaling AI solutions, sharing knowledge, and reducing capability asymmetries, particularly across platforms and supply chains (Li H. et al., 2025; Meinhold et al., 2025). Multi-actor coordination and collaborative governance are linked to higher innovation returns and technology transfer, although challenges remain around intellectual property and cultural alignment (Secundo et al., 2025; Zhou et al., 2025; Xue et al., 2025).
Ethical, governance, and implementation challenges of AI	18/50 Articles	Studies increasingly address privacy, bias, transparency, and accountability issues, proposing governance frameworks and responsible AI practices that strengthen organizational legitimacy (Dogru et al., 2025; Li H. et al., 2025). The need to integrate ethical criteria from design to strategic portfolio levels is emphasized, including XAI and socio-technical oversight mechanisms, particularly in international and sustainability contexts (Varriale et al., 2025; Meinhold et al., 2025; Chen and Zhang, 2024).
Role of AI in startups and entrepreneurial ecosystems	10/50 Articles	Evidence shows that startups leverage AI to accelerate learning, scale lightweight data models, and orchestrate alliances with platforms, while established firms hold advantages in resources, infrastructure, and standardization (Li H. et al., 2025; Almansour, 2024). Gaps persist in differential outcomes and adoption trajectories under financial and complementary capability constraints, as well as in governance and talent challenges (Carayannis et al., 2025; Khan et al., 2025; Muhammad et al., 2025).
AI in international business and global market dynamics	19/50 Articles	Research highlights AI’s contribution to optimizing global value chains, market intelligence, and cross-border coordination, with implications for geostrategic advantage and technological diplomacy (Dogru et al., 2025; Meinhold et al., 2025). At the same time, it notes regulatory heterogeneity and ethical standardization gaps that affect international scalability and data interoperability (Liu et al., 2025; Chen and Zhang, 2024; Zhou et al., 2025).
Integration of AI into business models and digital transformation	45/50 Articles	AI is established as the core of digital transformation by integrating with management systems (e.g., ERP/CRM, data platforms), enabling efficiency, personalization, and new revenue streams; its alignment with corporate objectives is crucial for value capture (Li H. et al., 2025; Naeem et al., 2025). The literature emphasizes the need for absorptive capacity, organizational redesign, and technological governance to sustain scaling (Varriale et al., 2025; Meinhold et al., 2025; Secundo et al., 2025).
Intellectual capital, investment, and strategic leadership in AI	23/50 Articles	Knowledge management and strategically oriented investment are associated with greater innovative performance and resilience, especially when converging with human-AI leadership and organizational learning (Secundo et al., 2025; Almansour, 2024). Evidence suggests that the combination of intellectual capital, talent policies, and data governance mechanisms determines adoption maturity and competitive impact (Yang et al., 2024; Wang et al., 2025; Zhong et al., 2025).

The themes of governance, ethics, and algorithmic responsibility form a third current that underscores the need for transparent normative frameworks. Simultaneously, studies on collaboration and interorganizational alliances highlight the relevance of associations between companies, governments, and universities in the expansion of AI use.

The adoption of AI in startups, along with digital transformation in established corporations, illustrates gaps in resources, agility, and infrastructure. Finally, emerging themes revolve around human-AI leadership, intellectual capital, and strategic investment in AI, configuring a business ecosystem where technological innovation and sustainability converge as central axes of global competitiveness.

## 5 Discussion

### 5.1 General bibliometric landscape and evolution of research

The bibliometric analysis reveals an exponential growth in scientific production on artificial intelligence (AI) and business strategy between 2016 and 2025. As observed in Figure 1, the publication curve evidences an acceleration after 2020, coinciding with the maturity of advanced analytics and the emergence of generative AI. This pattern reflects the growing centrality of AI in strategic planning and innovation management on a global scale.

The patterns identified through the bibliometric and qualitative analyses can be interpreted through several complementary theoretical lenses that elucidate the mechanisms by which artificial intelligence contributes to organizational value creation.

From the standpoint of the Resource-Based View (RBV), AI technologies may be conceptualized as strategic resources that bolster firms’ capacity to generate sustained competitive advantage, provided they are valuable, rare, inimitable, and organizationally embedded. In this framework, data infrastructures, machine learning competencies, and advanced analytical capabilities constitute core strategic assets that enable organizations to process vast quantities of information and inform high-level strategic decision-making.

Nevertheless, possession of such technological resources does not, in itself, guarantee superior performance. Extant literature underscores that the realization of AI’s strategic value hinges critically on firms’ dynamic capabilities—the organizational processes through which technological resources are sensed, seized, integrated, reconfigured, and redeployed in response to evolving environmental conditions. Consequently, AI adoption emerges as an iterative process of organizational learning, through which firms cultivate specialized capabilities in areas such as data governance, digital transformation leadership, and adaptive strategic formulation.

Concurrently, the diffusion and strategic deployment of AI are profoundly influenced by the institutional environment, encompassing regulatory regimes, industry norms, and broader societal expectations concerning transparency, fairness, and accountability in algorithmic decision systems. Institutional theory illuminates the ways in which organizations conform to—or strategically negotiate—external isomorphic pressures, including compliance mandates, ethical standards, and stakeholder demands for responsible AI practices.

Complementing these perspectives, the burgeoning literature on algorithmic governance offers a further interpretive framework for understanding how organizations mitigate the risks inherent in automated decision-making. Effective governance mechanisms—encompassing data quality assurance, algorithmic explainability and auditability, ethical review processes, and robust accountability structures—are indispensable for ensuring that AI systems deliver strategic benefits while preserving organizational legitimacy and maintaining stakeholder trust.

Collectively, these theoretical perspectives indicate that the strategic impact of artificial intelligence is not determined solely by the adoption of technological solutions. Rather, it arises from the synergistic interplay among AI-specific capabilities, organizational dynamic capabilities, and contextual governance arrangements, which together determine the performance outcomes of AI-enabled strategies.

The distribution of countries and authors confirms a polycentric and cooperative structure of the field. Figure 2 shows an international co-authorship network dominated by institutions from Europe, Asia, and North America, with significant connections between the United Kingdom, China, the United States, and India. This framework reinforces the interdisciplinary and transnational nature of the research, where data sciences, management, digital economy, and technological governance converge.

The keyword and thematic cluster analysis (Figure 4) reveals five interconnected research cores: (1) AI and machine learning, (2) digital transformation, (3) strategy and value creation, (4) sustainability and responsible innovation, and (5) generative models and algorithmic ethics. Finally, Figure 4 shows an emerging cluster with terms such as ChatGPT, large language models, and explainable AI, which evidences a new investigative frontier focused on transparency, biases, and automated decisions.

These bibliometric results confirm that the literature has evolved from a technological perspective toward a strategic-organizational vision, where AI is conceived as an intangible asset that redefines the competitiveness and sustainability of global companies.

### 5.2 Strategic integration of artificial intelligence

The findings evidence a solid consensus around the role of artificial intelligence (AI) as a catalyst for strategic transformation. The most cited studies (Dogru et al., 2025; Li H. et al., 2025; Naeem et al., 2025; Secundo et al., 2025) agree that AI strengthens planning capacity, strategy formulation, and decision-making through predictive analytics and machine learning, improving precision and mitigating risks in uncertain environments. This finding supports the integration of AI with consolidated management frameworks such as dynamic capabilities and the resource-based view, which position technology as a central strategic asset.

From a theoretical standpoint, these findings can be interpreted through the lens of the Resource-Based View (RBV). Within this framework, firms attain sustainable competitive advantage by possessing resources that are valuable, rare, inimitable, and non-substitutable (VRIN). The literature reviewed indicates that artificial intelligence increasingly functions as such a strategic resource when integrated with organizational data assets, analytical competencies, and complementary digital infrastructures. Multiple studies demonstrate that AI-driven analytics augment firms’ ability to generate actionable insights, optimize operational processes, and uncover novel market opportunities, thereby reinforcing the notion that data and algorithmic capabilities constitute core strategic assets in contemporary digital ecosystems.

Nevertheless, the evidence also underscores that AI, in isolation, does not automatically confer competitive advantage. Its strategic value is contingent upon the manner in which firms orchestrate the integration of technological resources with established organizational routines, governance structures, and human expertise. Effective realization of AI’s potential thus requires deliberate alignment between technological affordances and internal organizational capabilities.

Notwithstanding the predominantly optimistic discourse surrounding AI adoption, the reviewed literature simultaneously reveals notable contradictions and persistent theoretical and empirical debates. While a substantial body of research emphasizes AI’s capacity to drive productivity gains and foster innovation, other contributions draw attention to significant risks, including algorithmic bias, governance deficiencies, and the progressive erosion of stakeholder trust. For instance, certain empirical studies report that AI-supported decision systems enhance strategic efficiency and decisional speed, whereas others document that over-reliance on automated analytics may diminish managerial discretion, provoke employee resistance, or generate opposition among external stakeholders. Likewise, although AI-facilitated digital transformation is often linked to superior sustainability outcomes, evidence from select sectors points to potential trade-offs between short-term technological efficiencies and longer-term social or environmental objectives.

Collectively, these tensions indicate that the relationship between AI adoption and organizational performance is neither linear nor universal, but rather contingent upon a range of contextual moderators—including the robustness of governance frameworks, the characteristics of the institutional environment, and the prevailing features of organizational culture.

However, differences between sectors and degrees of digital maturity generate disparate results. Some studies emphasize that the strategic adoption of AI remains partial or focused on operational tasks (Wu et al., 2025; Ayala et al., 2025), which limits its transformative impact. These divergences reflect the gap between the theoretical potential of AI and its practical implementation, conditioned by data quality, technological infrastructure, and organizational culture.

### 5.3 Innovation, value creation, and competitive advantage

The reviewed literature holds that AI drives innovation and value creation at multiple levels. The studies by Li H. et al. (2025), Naeem et al. (2025), and Fosso Wamba et al. (2025) show that AI favors the development of intelligent products, service personalization, and optimization of data-based business models. Likewise, the synergy between AI and open innovation strategies allows accelerating knowledge transfer and reducing uncertainty in global competitive environments.

However, divergence persists regarding the sustainability of these results. While Dogru et al. (2025) and Secundo et al. (2025) highlight measurable increases in performance and innovation, other authors (Varriale et al., 2025; Meinhold et al., 2025) point out that long-term strategic benefits depend on the alignment between corporate objectives and digital capabilities. In this sense, AI-driven value creation is not an automatic result of technological adoption, but of the effective integration between human capital, data, and organizational leadership.

Despite the predominant emphasis in the literature on the positive contributions of artificial intelligence to organizational efficiency, agility, and competitive advantage, the present review also uncovers significant tensions and limitations associated with AI adoption in organizational settings. A number of studies document instances in which AI implementations have yielded limited—or, in some cases, negative—returns on investment, particularly when organizations possess inadequate data infrastructure, insufficient managerial expertise, or poor alignment between AI initiatives and existing strategic processes. Under such conditions, the introduction of AI technologies may paradoxically increase operational complexity without delivering commensurate strategic gains.

Moreover, the literature increasingly draws attention to concerns surrounding algorithmic bias and unintended discriminatory outcomes, particularly when machine learning models are trained on incomplete, unrepresentative, or historically biased datasets. These issues pose profound ethical and governance challenges for organizations seeking to deploy AI-supported decision systems responsibly.

An additional area of emerging debate pertains to the risks of excessive automation in strategic processes. Although automated analytics can enhance decisional velocity and information-processing capacity, over-reliance on algorithmic outputs may diminish managerial discretion, constrain organizational learning processes, or engender resistance among employees and external stakeholders.

Furthermore, the progressive expansion of AI-driven decision-making has attracted heightened regulatory and societal scrutiny, prompting the development of new governance frameworks designed to promote transparency, accountability, and ethical oversight of algorithmic systems. These institutional developments indicate that the strategic value of AI is contingent not merely upon technological sophistication, but also upon organizations’ capacity to establish robust governance structures capable of reconciling innovation imperatives with responsible implementation.

Taken collectively, these findings suggest that the impact of artificial intelligence on organizational performance is contingent rather than uniformly positive. It hinges on the complex interplay among technological capabilities, governance mechanisms, organizational readiness, and broader institutional environments.

These insights can also be fruitfully interpreted through the theoretical lens of dynamic capabilities, which underscores firms’ ability to sense emerging opportunities, seize them effectively, and reconfigure internal resources and routines in response to turbulent environmental conditions. In the specific context of AI adoption, dynamic capabilities manifest in organizations’ proficiency at assimilating novel analytical tools, redesigning decision-making architectures, and recalibrating business models to align with data-driven paradigms. Empirical contributions within the reviewed literature consistently indicate that firms exhibiting higher levels of digital maturity and adaptive learning capabilities are better positioned to realize strategic transformation through AI. This evidence reinforces the argument that the benefits of AI adoption are not determined exclusively by the scale of technological investment, but rather by the organization’s sustained ability to adapt structures, develop requisite competencies, and realign strategic orientations in evolving technological landscapes.

### 5.4 Ethical challenges and algorithmic governance

The ethical dimension emerges as a transversal line of research. Recent studies emphasize the relevance of privacy, transparency, and mitigation of algorithmic bias as pillars of AI governance (Dogru et al., 2025; Li et al., 2025; Jin and Ryu, 2025; Fosso Wamba et al., 2025). The appearance of the Explainable AI (XAI) concept in the most recent clusters (Figure 4) reflects the need to make automated decisions comprehensible and strengthen public trust.

However, the practical application of ethical governance varies between regions and industries. Studies such as those by Mahajan et al. (2025) and Attah et al. (2024) highlight significant regulatory gaps, especially in emerging economies where institutional maturity is limited. This regulatory heterogeneity explains the differences in the depth and effectiveness of adopted ethical frameworks. Consequently, the literature suggests advancing toward the international harmonization of ethical standards and algorithmic audit mechanisms.

**Figure 5:**
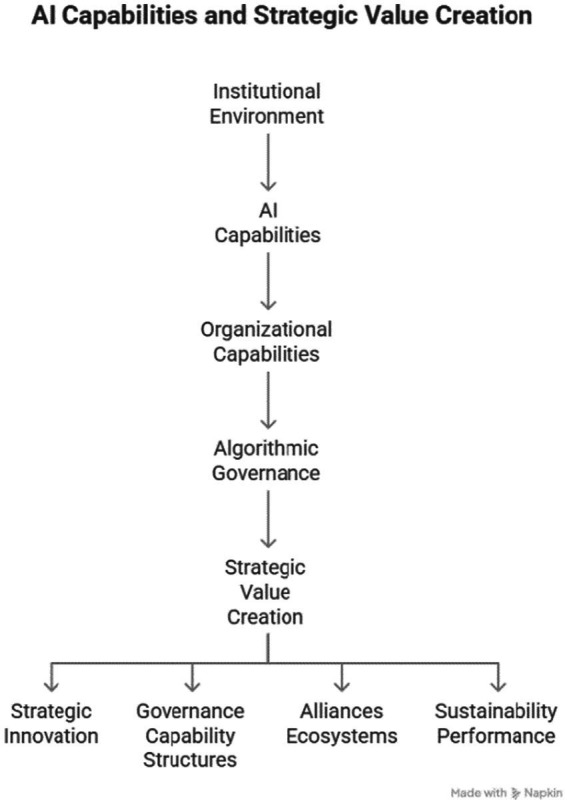
Conceptual framework linking AI capabilities, organizational capabilities, governance mechanisms, and strategic outcomes in AI-driven business environments.

Based on the integration of the bibliometric findings and the theoretical perspectives discussed above, a conceptual framework is proposed to illustrate how AI capabilities interact with organizational capabilities and governance conditions to generate strategic outcomes (see Figure 5).

This framework highlights that the value generated by AI technologies is contingent upon the alignment between technological capabilities, organizational competencies, and governance mechanisms that ensure responsible and effective deployment.

### 5.5 Dynamics of strategic alliances and interorganizational cooperation

AI not only reconfigures internal operations but also external relations between actors. The studies by Li H. et al. (2025), Meinhold et al. (2025), and Zhou et al. (2025) underscore that strategic alliances and innovation ecosystems are determinants for scaling AI solutions and sharing specialized knowledge.

Interorganizational cooperation facilitates access to talent, data infrastructure, and international markets, elements crucial for maintaining global competitiveness.

However, there are divergent views on the balance between collaboration and control. Some works (Cho and Reuer, 2023; Kaggwa et al., 2023) warn that technological dependence or lack of shared governance can limit the benefits of alliances. These tensions reflect the duality between the pursuit of open innovation and the need to protect intangible assets such as intellectual property or strategic knowledge.

### 5.6 Differences between startups and established companies

The results confirm that the organizational context conditions AI adoption. Startups, according to Li H. et al. (2025) and Almansour (2024), leverage AI to explore market niches and experiment with agile solutions, while established companies tend to scale and integrate technology into complex and standardized structures.

This distinction responds to differences in resources, innovation culture, and investment horizon. Even though startups face financial and talent restrictions, their ability to pivot quickly turns them into natural innovation laboratories. In contrast, consolidated organizations have greater analytical capacity, but their internal bureaucracy can slow down digital transformation. Thus, the reviewed studies show that value creation through AI depends as much on agility and adaptive leadership as on the available technical infrastructure.

### 5.7 Digital transformation, sustainability, and global competitiveness

AI positions itself as the backbone axis of digital transformation and corporate sustainability. More than 80% of the analyzed studies link AI with operational efficiency, process personalization, and reduction of environmental impact (Li H. et al., 2025; Naeem et al., 2025; Varriale et al., 2025; Meinhold et al., 2025; Secundo et al., 2025). Recent literature introduces the relationship between AI and ESG (Environmental, Social, Governance) objectives, highlighting its capacity to optimize resource use, minimize waste, and strengthen transparency in the value chain.

However, regional and regulatory differences influence the adoption of sustainable AI. While Europe leads in regulation and ethical practices, Latin America and Asia present uneven advances. These asymmetries suggest that the global competitiveness of AI will increasingly depend on the convergence between technological innovation and social responsibility.

### 5.8 Methodological considerations and projections for future research

Methodologically, the literature combines bibliometric approaches, case studies, and empirical analyses, which allows a comprehensive understanding of AI from different perspectives. This methodological diversity has enriched the field, although it also hinders comparison between studies and the accumulation of longitudinal evidence.

The detected limitations include the scarcity of temporal follow-up research and the thematic concentration in technological and manufacturing sectors, with less attention to educational, public, and social contexts. It is recommended that future research develop multiscale explanatory models that integrate human, ethical, and environmental dimensions, as well as international comparative studies that examine how AI governance impacts organizational sustainability.

In this way, the evidence suggests that the future of research on strategic AI must be oriented toward understanding its transformative value from an integral, interdisciplinary, and ethical perspective.

Overall, these findings indicate that the strategic value of artificial intelligence should be understood not as a purely technological phenomenon but as an organizational and institutional process shaped by resources, capabilities, governance structures, and broader ecosystem dynamics.

### 5.9 Citation window considerations

An important consideration in interpreting citation patterns is the influence of citation window effects, particularly for very recent publications. A substantial proportion of the studies included in the dataset were published between 2024 and 2025, which mirrors the accelerated growth of research at the intersection of artificial intelligence and strategic management in recent years. Nonetheless, citation counts for newly published articles must be interpreted cautiously, as these works have had comparatively little time to accumulate citations relative to earlier contributions.

To address potential citation window bias, the qualitative narrative synthesis did not rely solely on citation counts as the primary criterion for selecting influential studies. Rather, the identification of key works was guided by a dual assessment of citation impact and conceptual contribution to the strategic application and value creation potential of artificial intelligence within business contexts. This balanced approach enabled the inclusion of emerging, high-impact publications that are currently shaping the research agenda, while simultaneously preserving analytical equilibrium through the consideration of earlier foundational studies in the field.

Moreover, the bibliometric analysis in this study primarily seeks to delineate thematic structures, collaboration networks, and conceptual trajectories, rather than to assess the absolute maturity of the research domain on the basis of citation metrics alone. Accordingly, the prominence of recent publications should be understood as indicative of the dynamic, rapidly evolving character of AI-related inquiry in strategic management, rather than as evidence of an inflated perception of the field’s maturity.

## 6 Conclusion

The present study offers a comprehensive view of the role of artificial intelligence (AI) as a transformative axis in strategic management and global competitiveness. The bibliometric analysis of scientific production (2016–2025) reveals a sustained expansion and a process of thematic consolidation around five research cores: (1) strategic integration of AI, (2) innovation and value creation, (3) ethical governance, (4) strategic alliances, and (5) digital sustainability. This multidimensional structure reflects the transition from a predominantly technical approach toward a strategic and organizational vision, where AI is configured as a key resource for data-based decision-making, resilience, and business sustainability.

The qualitative findings from the 50 most cited articles confirm that AI strengthens operational efficiency, innovation, and adaptive capacity. The studies by Dogru et al. (2025), Li H. et al. (2025), Naeem et al. (2025), and Secundo et al. (2025) agree that the integration of AI with consolidated management frameworks—such as resource and capabilities theory or dynamic capabilities—reinforces sustainable competitive advantage. However, important gaps persist in its practical implementation, especially in data governance, algorithmic ethics, and the institutional preparedness of companies.

Likewise, the bibliometric analysis identifies a polycentric global collaboration network, dominated by universities and technological centers from Europe, Asia, and North America, which act as poles of knowledge generation and innovation. This pattern evidences the growing interdependence between science, industry, and public policy in shaping the global AI ecosystem. Finally, the emerging cluster of terms such as ChatGPT, large language models, and explainable AI anticipates a new research frontier, centered on interpretability, biases, and the ethical implications of generative models, which will mark the scientific agenda of the coming years.

Together, the results of this study allow concluding that AI has evolved from being a technological tool to consolidating itself as a strategic asset of global value, whose impact transcends operational efficiency and redefines organizational structures, inter-institutional alliances, and sustainable business models.

### 6.1 Practical implications

The results of the study offer several relevant implications for business management, policy formulation, and organizational innovation:

- Strategic management and digital leadership: Companies must incorporate AI into their planning and decision-making processes, not only as technical support, but as a strategic instrument that enhances the corporate vision. This requires strengthening digital leadership and data literacy among executive teams.
- Ethical and regulatory governance: The responsible implementation of AI demands policies of transparency, bias mitigation, and algorithmic audits. Organizations must adopt Explainable AI frameworks to ensure accountability and user trust.
- Innovation and sustainability: AI can be integrated into corporate sustainability strategies, optimizing energy consumption, reducing waste, and fostering circular supply chains. In this way, it aligns with the Sustainable Development Goals (SDGs) and ESG (Environmental, Social, Governance) criteria.
- Collaborative ecosystems: Alliances between startups, corporations, and academic institutions are essential to accelerate innovation and share knowledge. Public-private cooperation policies can facilitate technology transfer and reduce regional digital gaps.
- Organizational capabilities: Successful AI adoption requires developing internal capabilities in advanced analytics, data management, and adaptive leadership. Companies must invest in training and knowledge governance as structural elements of their competitiveness.

These implications evidence that AI not only transforms business processes but also the forms of leadership, learning, and corporate social responsibility.

### 6.2 Future areas of research

From the bibliometric trends and the gaps detected in the literature, several promising lines of research are identified:

- Longitudinal evaluation of AI impact: Future studies should analyze how AI adoption influences company performance and sustainability over time, through longitudinal and intersectoral comparative designs.
- Integration between AI and global sustainability: It is necessary to deepen the relationship between AI, green innovation, and environmental governance, evaluating how algorithms can contribute to the transition toward low-carbon economies.
- Algorithmic governance and ethics in emerging contexts: Research is required on the implementation of ethical frameworks in regions with low regulatory capacity, particularly in Latin America and Africa, where AI adoption is expanding but lacks solid regulation.
- Generative AI and human capital: The rise of generative models (e.g., ChatGPT) raises questions about productivity, creativity, and human talent management. Future research must explore their responsible integration in work environments.
- Hybrid human-algorithmic decision models: Another emerging area consists of analyzing how human decisions interact with AI recommendations, especially in sensitive sectors such as health, education, or public administration.
- AI-driven global value chains: It is suggested to expand the analysis of how AI redefines international relations and knowledge flows, affecting the distribution of power and global competitiveness.

These directions point to an interdisciplinary research agenda that combines empirical analysis, applied ethics, digital governance, and sustainability, to build a more holistic understanding of the strategic value of artificial intelligence in contemporary society.

## Data Availability

The original contributions presented in the study are included in the article/supplementary material, further inquiries can be directed to the corresponding author.
