# Supplementary material for: Strategic value driven by artificial intelligence in global businesses: a bibliometric and qualitative analysis of the most influential literature
Source: Front Artif Intell. 2026 Apr 13;9:1800412. doi: 10.3389/frai.2026.1800412 (PMC13111250; doi:10.3389/frai.2026.1800412)
Supplement: Supplementary file 1 [file Table_1.docx]

Annex 1 Thematic Comparative Summary of Key Studies on AI-Driven Strategic Value (2016–2025)

| Study | AI Integration into Strategy | Innovation and Value Creation | Ethical and Governance Challenges | Strategic Alliance Dynamics | AI Use in Startups vs Established Firms |
| --- | --- | --- | --- | --- | --- |
| Dogru et al., 2025 | AI integrates predictive analytics into planning and decision‑making; strengthens strategic formulation. | AI drives product/service innovation and new data‑driven business models. | Addresses privacy, bias, transparency, and responsible AI governance. | Main focus on the firm; strategic alliances are marginal. | Mixed application: startups explore; incumbents scale and standardize. |
| Y. Li et al., 2025 | AI integrates predictive analytics into planning and decision‑making; strengthens strategic formulation. | AI drives product/service innovation and new data‑driven business models. | Addresses privacy, bias, transparency, and responsible AI governance. | Analyzes alliances/ecosystems and interorganizational coordination to scale AI. | Mixed application: startups explore; incumbents scale and standardize. |
| Naeem et al., 2025 | AI integrates predictive analytics into planning and decision‑making; strengthens strategic formulation. | AI drives product/service innovation and new data‑driven business models. | Addresses privacy, bias, transparency, and responsible AI governance. | Reports focused collaboration and co‑creation with technology actors. | Mixed application: startups explore; incumbents scale and standardize. |
| Varriale et al., 2025 | Discusses AI uses with general strategic implications. | AI impacts on value creation are underdeveloped. | Addresses privacy, bias, transparency, and responsible AI governance. | Main focus on the firm; strategic alliances are marginal. | Mixed application: startups explore; incumbents scale and standardize. |
| Meinhold et al., 2025 | AI integrates predictive analytics into planning and decision‑making; strengthens strategic formulation. | AI impacts on value creation are underdeveloped. | Addresses privacy, bias, transparency, and responsible AI governance. | Analyzes alliances/ecosystems and interorganizational coordination to scale AI. | Mixed application: startups explore; incumbents scale and standardize. |
| Secundo et al., 2025 | AI integrates predictive analytics into planning and decision‑making; strengthens strategic formulation. | AI drives product/service innovation and new data‑driven business models. | Ethical and governance dimension is not central in the study. | Analyzes alliances/ecosystems and interorganizational coordination to scale AI. | Mixed application: startups explore; incumbents scale and standardize. |
| Liu et al., 2025 | Discusses AI uses with general strategic implications. | AI drives product/service innovation and new data‑driven business models. | Ethical and governance dimension is not central in the study. | Main focus on the firm; strategic alliances are marginal. | Focused on established firms: corporate orchestration and exploitation of data economies. |
| Chen & Zhang, 2024 | Discusses AI uses with general strategic implications. | AI drives product/service innovation and new data‑driven business models. | Addresses privacy, bias, transparency, and responsible AI governance. | Main focus on the firm; strategic alliances are marginal. | Focused on established firms: corporate orchestration and exploitation of data economies. |
| Almansour, 2025 | AI integrates predictive analytics into planning and decision‑making; strengthens strategic formulation. | AI drives product/service innovation and new data‑driven business models. | Ethical and governance dimension is not central in the study. | Main focus on the firm; strategic alliances are marginal. | Focused on startups/SMEs: agility, experimentation, and scaling with limited resources. |
| Zhou et al., 2025 | Discusses AI uses with general strategic implications. | AI drives product/service innovation and new data‑driven business models. | Addresses privacy, bias, transparency, and responsible AI governance. | Analyzes alliances/ecosystems and interorganizational coordination to scale AI. | Focused on established firms: corporate orchestration and exploitation of data economies. |
| Yang et al., 2024 | AI integrates predictive analytics into planning and decision‑making; strengthens strategic formulation. | AI drives product/service innovation and new data‑driven business models. | Ethical and governance dimension is not central in the study. | Main focus on the firm; strategic alliances are marginal. | Focused on established firms: corporate orchestration and exploitation of data economies. |
| S. Wang & Zhang, 2025ª | AI optimizes processes and forecasting and has implications for corporate strategy. | AI drives product/service innovation and new data‑driven business models. | Addresses privacy, bias, transparency, and responsible AI governance. | Main focus on the firm; strategic alliances are marginal. | Mixed application: startups explore; incumbents scale and standardize. |
| Carayannis et al., 2025 | AI integrates predictive analytics into planning and decision‑making; strengthens strategic formulation. | AI improves efficiency and organizational learning with effects on value creation. | Points to risks, compliance, and regulatory challenges associated with AI. | Main focus on the firm; strategic alliances are marginal. | Focused on startups/SMEs: agility, experimentation, and scaling with limited resources. |
| Y. Wang et al., 2025 | AI integrates predictive analytics into planning and decision‑making; strengthens strategic formulation. | AI drives product/service innovation and new data‑driven business models. | Addresses privacy, bias, transparency, and responsible AI governance. | Main focus on the firm; strategic alliances are marginal. | Focused on established firms: corporate orchestration and exploitation of data economies. |
| Tortorella et al., 2025 | AI integrates predictive analytics into planning and decision‑making; strengthens strategic formulation. | AI impacts on value creation are underdeveloped. | Addresses privacy, bias, transparency, and responsible AI governance. | Main focus on the firm; strategic alliances are marginal. | Mixed application: startups explore; incumbents scale and standardize. |
| Xue et al., 2025 | AI integrates predictive analytics into planning and decision‑making; strengthens strategic formulation. | AI drives product/service innovation and new data‑driven business models. | Addresses privacy, bias, transparency, and responsible AI governance. | Analyzes alliances/ecosystems and interorganizational coordination to scale AI. | Focused on established firms: corporate orchestration and exploitation of data economies. |
| Zhong et al., 2025 | Discusses AI uses with general strategic implications. | AI drives product/service innovation and new data‑driven business models. | Ethical and governance dimension is not central in the study. | Analyzes alliances/ecosystems and interorganizational coordination to scale AI. | Focused on established firms: corporate orchestration and exploitation of data economies. |
| Koponen et al., 2025 | Discusses AI uses with general strategic implications. | AI impacts on value creation are underdeveloped. | Ethical and governance dimension is not central in the study. | Main focus on the firm; strategic alliances are marginal. | Mixed application: startups explore; incumbents scale and standardize. |
| Lokanan & Maddhesia, 2025 | AI optimizes processes and forecasting and has implications for corporate strategy. | AI drives product/service innovation and new data‑driven business models. | Points to risks, compliance, and regulatory challenges associated with AI. | Analyzes alliances/ecosystems and interorganizational coordination to scale AI. | Mixed application: startups explore; incumbents scale and standardize. |
| Khan et al., 2025 | AI integrates predictive analytics into planning and decision‑making; strengthens strategic formulation. | AI drives product/service innovation and new data‑driven business models. | Addresses privacy, bias, transparency, and responsible AI governance. | Analyzes alliances/ecosystems and interorganizational coordination to scale AI. | Focused on startups/SMEs: agility, experimentation, and scaling with limited resources. |
| Bevilacqua et al., 2025 | AI integrates predictive analytics into planning and decision‑making; strengthens strategic formulation. | AI drives product/service innovation and new data‑driven business models. | Ethical and governance dimension is not central in the study. | Reports focused collaboration and co‑creation with technology actors. | Mixed application: startups explore; incumbents scale and standardize. |
| Muhammad et al., 2025 | Discusses AI uses with general strategic implications. | AI impacts on value creation are underdeveloped. | Ethical and governance dimension is not central in the study. | Analyzes alliances/ecosystems and interorganizational coordination to scale AI. | Focused on startups/SMEs: agility, experimentation, and scaling with limited resources. |
| H. Li et al., 2025 | AI optimizes processes and forecasting and has implications for corporate strategy. | AI drives product/service innovation and new data‑driven business models. | Points to risks, compliance, and regulatory challenges associated with AI. | Analyzes alliances/ecosystems and interorganizational coordination to scale AI. | Mixed application: startups explore; incumbents scale and standardize. |
| Parra‑López et al., 2025 | AI integrates predictive analytics into planning and decision‑making; strengthens strategic formulation. | AI drives product/service innovation and new data‑driven business models. | Points to risks, compliance, and regulatory challenges associated with AI. | Analyzes alliances/ecosystems and interorganizational coordination to scale AI. | Mixed application: startups explore; incumbents scale and standardize. |
| Modgil et al., 2025 | AI integrates predictive analytics into planning and decision‑making; strengthens strategic formulation. | AI drives product/service innovation and new data‑driven business models. | Points to risks, compliance, and regulatory challenges associated with AI. | Main focus on the firm; strategic alliances are marginal. | Mixed application: startups explore; incumbents scale and standardize. |
| Schwaeke et al., 2025 | AI integrates predictive analytics into planning and decision‑making; strengthens strategic formulation. | AI drives product/service innovation and new data‑driven business models. | Ethical and governance dimension is not central in the study. | Main focus on the firm; strategic alliances are marginal. | Focused on established firms: corporate orchestration and exploitation of data economies. |
| Malhotra & Kharub, 2024 | AI integrates predictive analytics into planning and decision‑making; strengthens strategic formulation. | AI drives product/service innovation and new data‑driven business models. | Ethical and governance dimension is not central in the study. | Analyzes alliances/ecosystems and interorganizational coordination to scale AI. | Mixed application: startups explore; incumbents scale and standardize. |
| Ma et al., 2025 | AI integrates predictive analytics into planning and decision‑making; strengthens strategic formulation. | AI drives product/service innovation and new data‑driven business models. | Ethical and governance dimension is not central in the study. | Main focus on the firm; strategic alliances are marginal. | Focused on established firms: corporate orchestration and exploitation of data economies. |
| Farmanesh et al., 2025 | AI integrates predictive analytics into planning and decision‑making; strengthens strategic formulation. | AI drives product/service innovation and new data‑driven business models. | Ethical and governance dimension is not central in the study. | Main focus on the firm; strategic alliances are marginal. | Focused on startups/SMEs: agility, experimentation, and scaling with limited resources. |
| Huang et al., 2025 | Discusses AI uses with general strategic implications. | AI drives product/service innovation and new data‑driven business models. | Ethical and governance dimension is not central in the study. | Main focus on the firm; strategic alliances are marginal. | Focused on established firms: corporate orchestration and exploitation of data economies. |
| S. Wang & Zhang, 2025c | Discusses AI uses with general strategic implications. | AI drives product/service innovation and new data‑driven business models. | Addresses privacy, bias, transparency, and responsible AI governance. | Main focus on the firm; strategic alliances are marginal. | Focused on startups/SMEs: agility, experimentation, and scaling with limited resources. |
| M. Wang et al., 2023 | AI integrates predictive analytics into planning and decision‑making; strengthens strategic formulation. | AI drives product/service innovation and new data‑driven business models. | Ethical and governance dimension is not central in the study. | Main focus on the firm; strategic alliances are marginal. | Mixed application: startups explore; incumbents scale and standardize. |
| Rainer Jr. et al., 2025 | AI optimizes processes and forecasting and has implications for corporate strategy. | AI impacts on value creation are underdeveloped. | Ethical and governance dimension is not central in the study. | Analyzes alliances/ecosystems and interorganizational coordination to scale AI. | Mixed application: startups explore; incumbents scale and standardize. |
| Sandeep et al., 2025 | AI integrates predictive analytics into planning and decision‑making; strengthens strategic formulation. | AI drives product/service innovation and new data‑driven business models. | Addresses privacy, bias, transparency, and responsible AI governance. | Analyzes alliances/ecosystems and interorganizational coordination to scale AI. | Focused on startups/SMEs: agility, experimentation, and scaling with limited resources. |
| Webster & Cain, 2024 | AI integrates predictive analytics into planning and decision‑making; strengthens strategic formulation. | AI impacts on value creation are underdeveloped. | Points to risks, compliance, and regulatory challenges associated with AI. | Main focus on the firm; strategic alliances are marginal. | Mixed application: startups explore; incumbents scale and standardize. |
| Pietsch & Mah, 2025 | Discusses AI uses with general strategic implications. | AI drives product/service innovation and new data‑driven business models. | Ethical and governance dimension is not central in the study. | Main focus on the firm; strategic alliances are marginal. | Mixed application: startups explore; incumbents scale and standardize. |
| Oprea & Bâra, 2025 | AI integrates predictive analytics into planning and decision‑making; strengthens strategic formulation. | AI drives product/service innovation and new data‑driven business models. | Ethical and governance dimension is not central in the study. | Analyzes alliances/ecosystems and interorganizational coordination to scale AI. | Mixed application: startups explore; incumbents scale and standardize. |
| Niu et al., 2025 | AI integrates predictive analytics into planning and decision‑making; strengthens strategic formulation. | AI improves efficiency and organizational learning with effects on value creation. | Ethical and governance dimension is not central in the study. | Main focus on the firm; strategic alliances are marginal. | Focused on established firms: corporate orchestration and exploitation of data economies. |
| Al‑Okaily, 2025 | AI integrates predictive analytics into planning and decision‑making; strengthens strategic formulation. | AI drives product/service innovation and new data‑driven business models. | Ethical and governance dimension is not central in the study. | Main focus on the firm; strategic alliances are marginal. | Focused on startups/SMEs: agility, experimentation, and scaling with limited resources. |
| Saura & Bužinskienė, 2025 | AI integrates predictive analytics into planning and decision‑making; strengthens strategic formulation. | AI drives product/service innovation and new data‑driven business models. | Addresses privacy, bias, transparency, and responsible AI governance. | Analyzes alliances/ecosystems and interorganizational coordination to scale AI. | Focused on startups/SMEs: agility, experimentation, and scaling with limited resources. |
| Ferk Savec & Jedrinović, 2025 | Discusses AI uses with general strategic implications. | AI drives product/service innovation and new data‑driven business models. | Points to risks, compliance, and regulatory challenges associated with AI. | Main focus on the firm; strategic alliances are marginal. | Mixed application: startups explore; incumbents scale and standardize. |
| Chandra & Feng, 2025 | AI integrates predictive analytics into planning and decision‑making; strengthens strategic formulation. | AI impacts on value creation are underdeveloped. | Addresses privacy, bias, transparency, and responsible AI governance. | Main focus on the firm; strategic alliances are marginal. | Mixed application: startups explore; incumbents scale and standardize. |
| Ghosh, 2025 | AI integrates predictive analytics into planning and decision‑making; strengthens strategic formulation. | AI drives product/service innovation and new data‑driven business models. | Ethical and governance dimension is not central in the study. | Main focus on the firm; strategic alliances are marginal. | Mixed application: startups explore; incumbents scale and standardize. |
| Gao et al., 2025 | AI integrates predictive analytics into planning and decision‑making; strengthens strategic formulation. | AI impacts on value creation are underdeveloped. | Addresses privacy, bias, transparency, and responsible AI governance. | Main focus on the firm; strategic alliances are marginal. | Focused on established firms: corporate orchestration and exploitation of data economies. |
| Oulefki et al., 2025 | AI integrates predictive analytics into planning and decision‑making; strengthens strategic formulation. | AI drives product/service innovation and new data‑driven business models. | Addresses privacy, bias, transparency, and responsible AI governance. | Analyzes alliances/ecosystems and interorganizational coordination to scale AI. | Mixed application: startups explore; incumbents scale and standardize. |
| S. Wang et al., 2025 | AI integrates predictive analytics into planning and decision‑making; strengthens strategic formulation. | AI drives product/service innovation and new data‑driven business models. | Ethical and governance dimension is not central in the study. | Main focus on the firm; strategic alliances are marginal. | Focused on established firms: corporate orchestration and exploitation of data economies. |
| Fosso Wamba et al., 2025 | AI integrates predictive analytics into planning and decision‑making; strengthens strategic formulation. | AI drives product/service innovation and new data‑driven business models. | Addresses privacy, bias, transparency, and responsible AI governance. | Analyzes alliances/ecosystems and interorganizational coordination to scale AI. | Focused on startups/SMEs: agility, experimentation, and scaling with limited resources. |
| Ayala et al., 2025 | AI optimizes processes and forecasting and has implications for corporate strategy. | AI drives product/service innovation and new data‑driven business models. | Ethical and governance dimension is not central in the study. | Main focus on the firm; strategic alliances are marginal. | Mixed application: startups explore; incumbents scale and standardize. |
| Jin & Ryu, 2025 | Discusses AI uses with general strategic implications. | AI drives product/service innovation and new data‑driven business models. | Addresses privacy, bias, transparency, and responsible AI governance. | Main focus on the firm; strategic alliances are marginal. | Focused on established firms: corporate orchestration and exploitation of data economies. |
| Wu et al., 2025 | AI optimizes processes and forecasting and has implications for corporate strategy. | AI drives product/service innovation and new data‑driven business models. | Ethical and governance dimension is not central in the study. | Analyzes alliances/ecosystems and interorganizational coordination to scale AI. | Focused on established firms: corporate orchestration and exploitation of data economies. |
